# In vivo toxicity and antioxidant of pressurize hot water *Phyllanthus tenellus* Roxb. extracts

**DOI:** 10.1186/s12906-021-03260-y

**Published:** 2021-03-09

**Authors:** Swee Keong Yeap, Chean Yeah Yong, Umar Faruq, Hui Kian Ong, Zahiah binti Mohamed Amin, Wan Yong Ho, Shaiful Sharifudin, Indu Bala Jaganath

**Affiliations:** 1 China-ASEAN College of Marine Sciences, Xiamen University Malaysia, 43900 Sepang, Selangor Malaysia; 2 Institute of Bioscience, Universiti Putra Malaysia, 43400 Serdang, Selangor Malaysia; 3 Faculty of Science, University of Nottingham Malaysia, Jalan Broga, 43500 Semenyih, Selangor Malaysia; 4 Biotechnology Research Centre, Malaysian Agricultural Research and Development Institute (MARDI), 43400 Serdang, Selangor Malaysia; 5 Strategic Planning & Innovation Management Centre, Malaysian Agricultural Research and Development Institute (MARDI), 43400 Serdang, Selangor Malaysia

**Keywords:** Pressurized, *Phyllanthus tenellus*, Hydrosable tannin, Toxicity, Antioxidant, Nitric oxide

## Abstract

**Background:**

*Phyllanthus tenellus* Roxb. has been traditionally used to treat inflammation and liver diseases and its medicinal property may be due to the presence of relatively high levels of hydrosable tannins. Recent report revealed that pressurized hot water extraction of *P. tenellus* significantly increased the concentration of hydrolysable tannins and its catabolites. Thus, this study was aimed to evaluate the in vivo toxicity and antioxidant capacity of pressurized hot water extraction of *P. tenellus* on healthy mice.

**Methods:**

Pressurized hot water extraction *P. tenellus* was carried out and standardized to 7.9% hydrosable tannins. In vitro toxicity of the extract was tested on NIH 3 T3 cell by MTT assay. The cellular antioxidant level was quantified by measuring cellular level of glutathione. Oral sub-chronic toxicity (200, 1000 and 3000 mg/kg body weight) of *P. tenellus* extract were evaluated on healthy mice. Liver and kidney antioxidant level was quantified by measuring levels of Ferric Reducing Antioxidant Potential (FRAP), superoxide dismutase, glutathione.

**Results:**

The *P. tenellus* extract did not induce cytotoxicity on murine NIH 3 T3 cells up to 200 μg/mL for 48 h. Besides, level of glutathione was higher in the extract treated NIH 3 T3 cells. *P. tenellus* extract did not cause mortality at all tested concentration. When treated with 1000 mg/kg of the extract, serum liver enzymes (ALP and ALT) and LDH were lower than normal control and mice treated with 200 mg/kg of extract. Moreover, SOD, FRAP and glutathione levels of liver of the mice treated with 200 and 1000 mg/kg of extract were higher than the normal control mice. On the other hand, when treated with 3000 mg/kg of extract, serum liver enzymes (ALP and ALT) and LDH were higher than normal mice without changing the liver SOD and glutathione level, which may contribute to the histological sign of ballooning hepatocyte.

**Conclusion:**

*P. tenellus* extract standardized with 7.9% hydrosable tannins and their catabolites increased the antioxidant levels while reducing the nitric oxide levels in both liver and kidney without causing any acute and sub-chronic toxicity in the mice.

## Background

The genus *Phyllanthus*, containing more than 700 species, is commonly used in traditional herbal remedies and is widely distributed throughout Asia and South America [1, 2]. In India, *Phyllanthus* has been used as a diuretic and for its antibacterial, antioxidant and immunomodulator properties [3]. *Phyllanthus tenellus* is less common compared to its close relative *P. niruri* and *P. amarus* but is considered an important species especially in the Brazilian tropical and sub-tropical regions. *P. tenellus* has been reported to contain tannins and ellagitannins [1], which is believed to contribute to its anti-hepatitis virus activity [4, 5], UV protection [6] and immunomodulatory effects [7]. *P. tenellus* extract was reported with in vitro [3, 8] antioxidant activity, which has contributed to its in vitro hepatoprotective effect [3]. In vivo study performed by Lee et al. [9] has supported the above findings where *P. tenellus* extract was able to strengthen the liver reduced glutathione peroxidase (GSH-Px) to promote recovery of carbon tetrachloride induced liver damage. Besides, *P. tenellus* extract was also reported with anti-inflammatory effect [7]. In addition, investigation have also revealed the antimicrobial activity of *P. tenellus* in comparison to other species of *Phyllanthus* [2]. Moreover, in vivo study has reported that *P. tenellus* exhibit potent analgesic effect against neurogenic and inflammatory pain [10, 11]. Unlike its more common relatives *P. niruri* and *P. amarus*, the bioactivities and safety studies of *P. tenellus* has been not well documented [12].

As the use of herbal medicines is expanding rapidly as alternative treatments worldwide, scientist have to ensure its safety through carrying out precise toxicity studies [2, 13]. Ethanol extract of aerial part of *P. tenellus* was previously reported with no toxic effect up to 2.5 g/kg body weight. Although *P. tenellus* ethanolic extract did not cause mortality up to 2.5 g/kg body weight, the treated rat was observed to have depressant behavior at this dosage [2]. This effect was correlated with the prolyl oligopeptidase and acetylcholinesterase inhibition by the corilagin present in the *P. tenellus* extract [14]. Our previous study has indicated that by modifying the extraction method and by just using pressurized water at 121 °C, we managed to significantly increase the concentration of bioactive metabolites particularly the hydrosable tannins, that believed to be Urolithin A that catabolized from ellagic acid [15] compared to previous report [1]. Besides, corilagin that was reported to cause depression [14] was not detected in the pressurized water extract [15]. As the chemical profile of this extract differs considerably from the previous report, there is a need to carry out safety and efficacy studies of the newly formulated *P. tenellus*. This study was therefore performed to evaluate the sub-chronic toxicity of *P. tenellus* extract. In addition, the antioxidant effect of the extract on healthy animals were also quantified in this study. Identification of the toxicity and antioxidant effect of the *P. tenellus* extract at the tested dosages help to select the range of concentration that suitable for the future in vivo bioactivities study.

## Methods

### Preparation of *P. tenellus* extract

The wild *P. tenellus* plant was sourced from Cameron Highlands, Malaysia, identified and verified by the taxonomist, Dr. Salmah Idris, from Malaysia Agricultural Research and Development Institute (MARDI). The plant has been deposited at MARDI’s herbarium under the botanical voucher (MD10525). Pressurized hot water extract of *Phyllanthus tenellus* was prepared according to the previous study. The extract utilized in this study was quantified by LC-MS.MS to standardize the concentration of the hydrosable tannins at approximate 8% [15]. *P. tenellus* extract was diluted in RPMI-1640 medium and distilled water at 1 g/mL for in vitro and in vivo assays, respectively.

### In vitro cytotoxicity and level of GSH of *P. tenellus* extract treated NIH 3 T3 cells

NIH 3 T3 (CRL-1658) was obtained from ATCC, USA and maintained in RPMI-1640 medium supplemented with 10% fetal bovine serum in 5% CO_2_ incubator. For the cytotoxicity test, 8 × 10^5^ cells/mL of NIH 3 T3 cells was seeded in 96 well plate overnight. On the next day, the seeded cells were treated with serial diluted *P. tenellus* extract (200, 100, 50, 25, 12.5, 6.25, 3.12 μg/mL) for 48 h. After the incubation period, each well was added with 20 μL of MTT solution. After 4 h of incubation, the supernatants were removed and the purple formazan crystal formed was dissolved with 100 μL dimethyl sulfoxide. The absorbance of the sample was measured at 570 nm using Quant ELISA plate reader (Bio-tek Instruments, USA). Percentage of viability of the extract treated cells was calculated by the absorbance ratio between extract treated cells with the untreated control cells multiplied by 100%.

For the quantification of GSH, NIH 3 T3 cells were treated with 200, 100, and 50 μg/mL. After 48 h of treatment, cells were collected and subjected repeated cycle of freezing by liquid nitrogen and thawing at 37 °C. Then, cell lysate was collected after centrifuging the cells extract at 13,000 rpm for 15 min at 4 °C. The GSH content was quantified by glutathione assay kit (Sigma-Aldrich, USA).

### Animal

This study was approved by the Animal Ethics Committee (AEC), Malaysian Agricultural Research and Development Institute (Approval no 20160902/R/MAEC00001) and compliance to the guidelines of the AEC, MARDI. Female BALB/c mice (*n* = 36; 6 weeks old) were purchased from animal house, Institute of Bioscience, Universiti Putra Malaysia. Mice (n = 3 per cage) were house in plastic cage and supplied with distilled water and standard pellets 702p (Gold Coin, Malaysia) ad libitum under 12 h of light/dark cycles per day with temperature ~ 22–24 °C and ~ 70% of humidity.

### Sub-chronic toxicity test

Sub-chronic toxicity test was designed and performed as previously reported [9]. After acclimatization, mice were randomly assigned into 4 groups (*n* = 9 per group). The control group received daily oral feeding of distilled water (200 μL) while *Phyllanthus* treated groups received daily oral treatments with 200, 1000 and 3000 mg/kg of *P. tenellus* extract dissolved in the distilled water for 28 days. Water and standard food pellets were provided ad libitum throughout the experimental period. After 28 days, weight of the mice was recorded. All mice were anesthetized with 2% isoflurane (1,349,014, Merck, USA) and sacrificed by cervical dislocation [16].. Serum, kidney, liver and spleen were collected and subjected to the following assays.

### Serum biochemical analysis

Level of serum liver enzyme markers aspartate aminotransferase (AST), alanine aminotransferase (ALT), alkaline phosphatase (ALP), creatinine and Lactic acid dehydrogenase (LDH) were quantified by Hitachi 902 Automatic Analyzer (Hitachi, Japan) using reagents from Roche (Germany).

### Liver and kidney antioxidant and nitric oxide quantification

Liver and kidney were collected from each mouse, washed with phosphate buffer saline (PBS) (Sigma-Aldrich, USA), weighed and meshed by 0.2 μm cell strainer (SPL Life Sciences, China) in cold PBS to obtain liver and kidney homogenate. The homogenate was subjected to ferric-reducing ability plasma (FRAP), superoxide dismutase (SOD), malondialdehyde (MDA) and nitric oxide (NO) quantification were based on the previously reported methods [17]. The GSH content was quantified by glutathione assay kit (Sigma-Aldrich, USA).

### Statistical analysis

Means and standard deviation from nine mice per groups (each with three technical replicates) were calculated. Significant differences (*p* < 0.05) between the normal and *P. tenellus* extract treated groups for all experiments were analyzed using one-way analysis of variance (ANOVA) followed by post-hoc Duncan analysis using SPSS version 20.

## Results

### In vitro cytotoxicity and glutathione level of *P. tenellus* extract

**Fig. 1:**
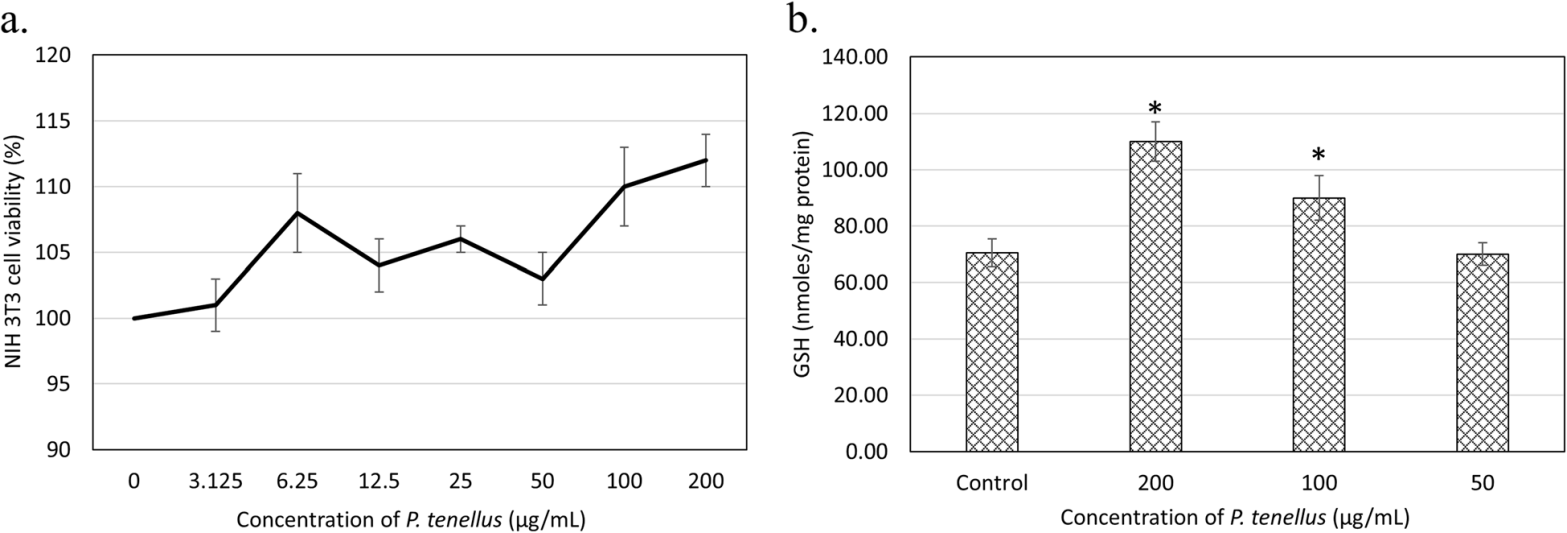
**a** Viability of NIH 3 T3 cells treated with *P. tenellus extract* (200, 100, 50, 25, 12.5, 6.25, 3.12 μg/mL) for 48 h **b** GSH content of NIH 3 T3 cells treated with *P. tenellus extract* (200, 100 and 50 μg/mL) for 48 h

*P. tenellus* extract did not reduce viability of NIH 3 T3 cells up to 200 μg/mL (Fig. 1a). For the viability assay, 200 and 100 μg/mL of the extract increased 10% of viability of the NIH 3 T3 cells comparing to the untreated group. Besides, 200 and 100 μg/mL of the extract also enhanced 1.5 and 1.3 folds of cellular GSH in NIH 3 T3 cells comparing to the untreated group (Fig. 1b).

### In vivo toxicity of *P. tenellus* extract

In this study, mice were orally fed with 200, 1000 and 3000 mg/kg body weight of pressurized hot water extract of *P. tenellus* for 28 days. Throughout the treatment period, all *P. tenellus* extract treated mice survived without showing any sign and symptoms of toxicity (Results not shown). In addition, no significant changes were observed for the final body weight, fasting glucose and organ/body weight ratio after 28 days of *P. tenellus* extract treatment (Table 1).

**Table 1:** Body weight (day 0 and day 28) and organ weight of control and *Phyllanthus* treated mice in sub-chronic toxicity study

	Control	***Phyllanthus*** 200	***Phyllanthus*** 1000	***Phyllanthus*** 3000
Day 0 BW (g)	19.85 ± 1.80	20.18 ± 1.60	19.89 ± 1.80	20.21 ± 1.40
Day 28 BW (g)	22.25 ± 2.20	22.33 ± 1.90	22.57 ± 1.70	22.14 ± 2.10
Fasting glucose (mmol/L)	5.21 ± 0.21	5.32 ± 0.44	5.17 ± 0.62	5.20 ± 0.32
Brain/BW ratio	0.014 ± 0.002	0.015 ± 0.003	0.014 ± 0.001	0.016 ± 0.002
Heart/BW ratio	0.006 ± 0.001	0.006 ± 0.001	0.005 ± 0.001	0.005 ± 0.001
Lung/BW ratio	0.008 ± 0.001	0.008 ± 0.001	0.008 ± 0.001	0.008 ± 0.001
Liver/BW ratio	0.061 ± 0.018	0.056 ± 0.013	0.046 ± 0.021	0.047 ± 0.008
Kidney/BW ratio	0.019 ± 0.010	0.017 ± 0.009	0.015 ± 0.013	0.016 ± 0.011
Spleen/BW ratio	0.005 ± 0.001	0.005 ± 0.001	0.004 ± 0.001	0.004 ± 0.001

### *P. tenellus* extract influenced serum biochemical markers and liver histology

**Fig. 2:**
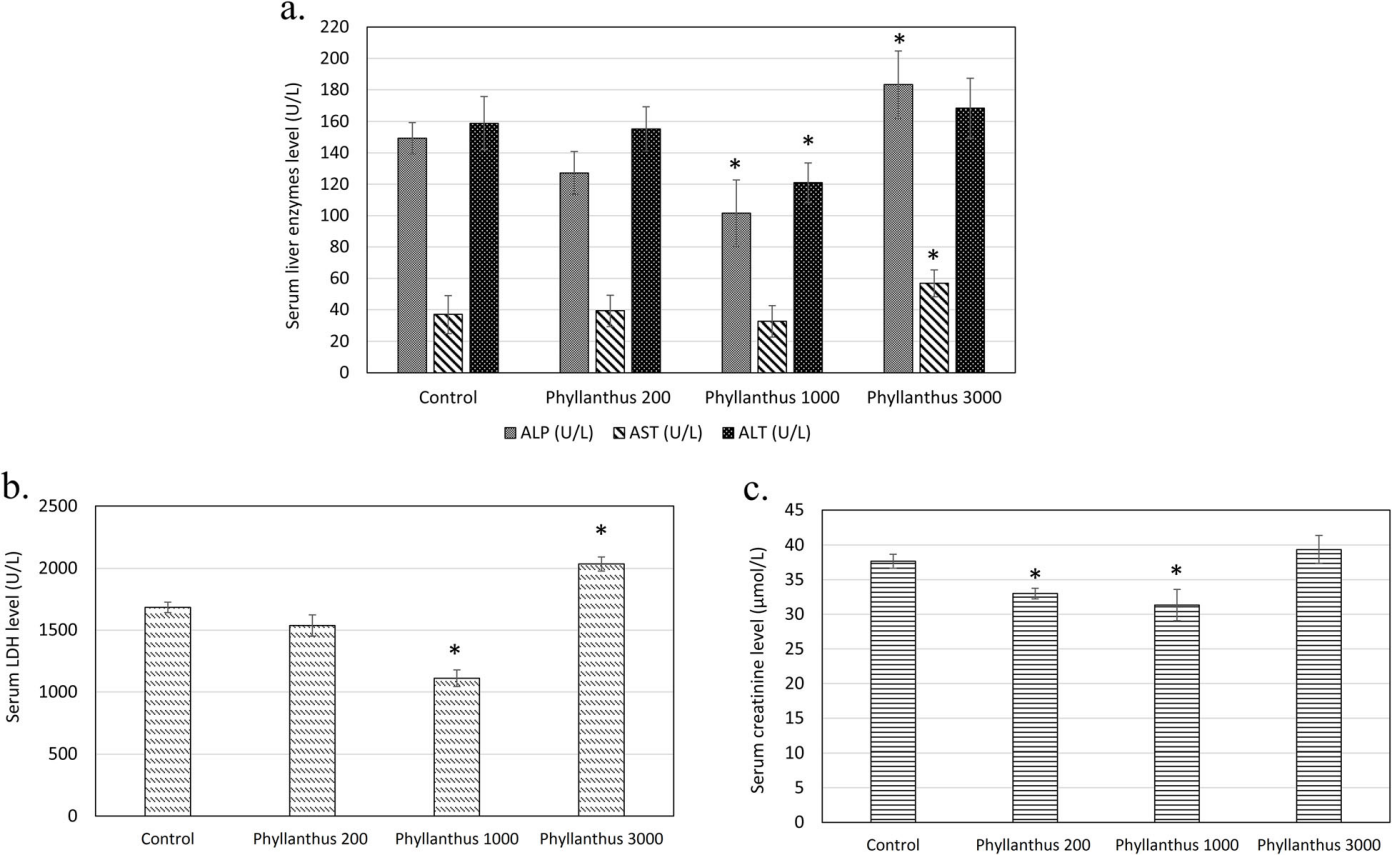
**a** Serum liver enzyme markers (ALP, AST and ALT); **b** Serum LDH; and **c** Serum creatinine levels of normal control and *Phyllanthus* (200, 1000 and 3000 mg/kg body weight) treated mice in sub-chronic toxicity study. *indicates a significant difference compared with the normal control group, *p* < 0.05

**Fig. 3:**
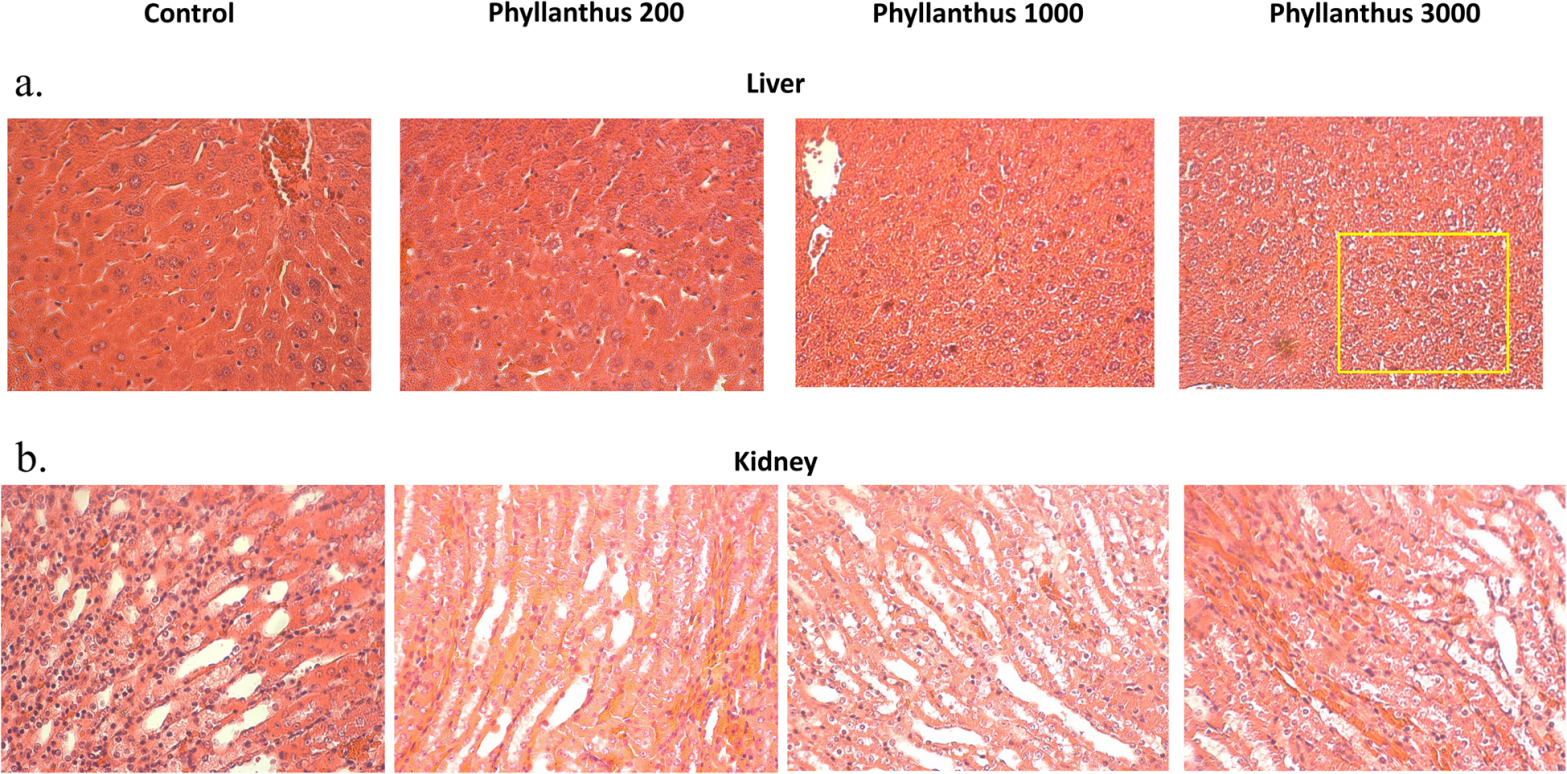
**a** Liver and **b** kidney histological section of normal control and *Phyllanthus* (200, 1000 and 3000 mg/kg body weight) treated mice in sub-chronic toxicity study. Yellow box indicates hepatocytes ballooning

Comparing to the normal control, 1000 mg/kg body weight of *P. tenellus* extract significantly (*p* < 0.05) reduced the serum ALP, ALT, LDH and creatinine levels, ie 0.85 folds, 0.98 folds and 0.91 folds compared to the control mice (Fig. 2a, b and c). On the other hand, 3000 mg/kg body weight of the extract was recorded with 1.23 folds, 1.53 folds and 1.2 folds of serum ALP, AST and LDH level (Fig. 2a and b) than the control mice. Unlike the serum liver enzyme, no significant changes of serum level of creatinine in the mice treated with 3000 mg/kg body weight of *P. tenellus* extract were observed compared to the untreated normal control mice (Fig. 2c). This result was supported by the histological assessment where ballooning was observed in the liver section of mice treated with 3000 mg/kg body weight of *P. tenellus* extract (Fig. 3a) without significant changes were observed in the kidney section (Fig. 3b). Moreover, no histological changes were observed in the liver and kidney section of mice treated with 200 and 1000 mg/kg body weight of *P. tenellus* extract.

### *P. tenellus* extract enhanced the liver and kidney antioxidant level

*P. tenellus* extract (1000 mg/kg body weight) enhanced the 1.8 folds, 1.6 folds, 1.7 folds of liver.

**Fig. 4:**
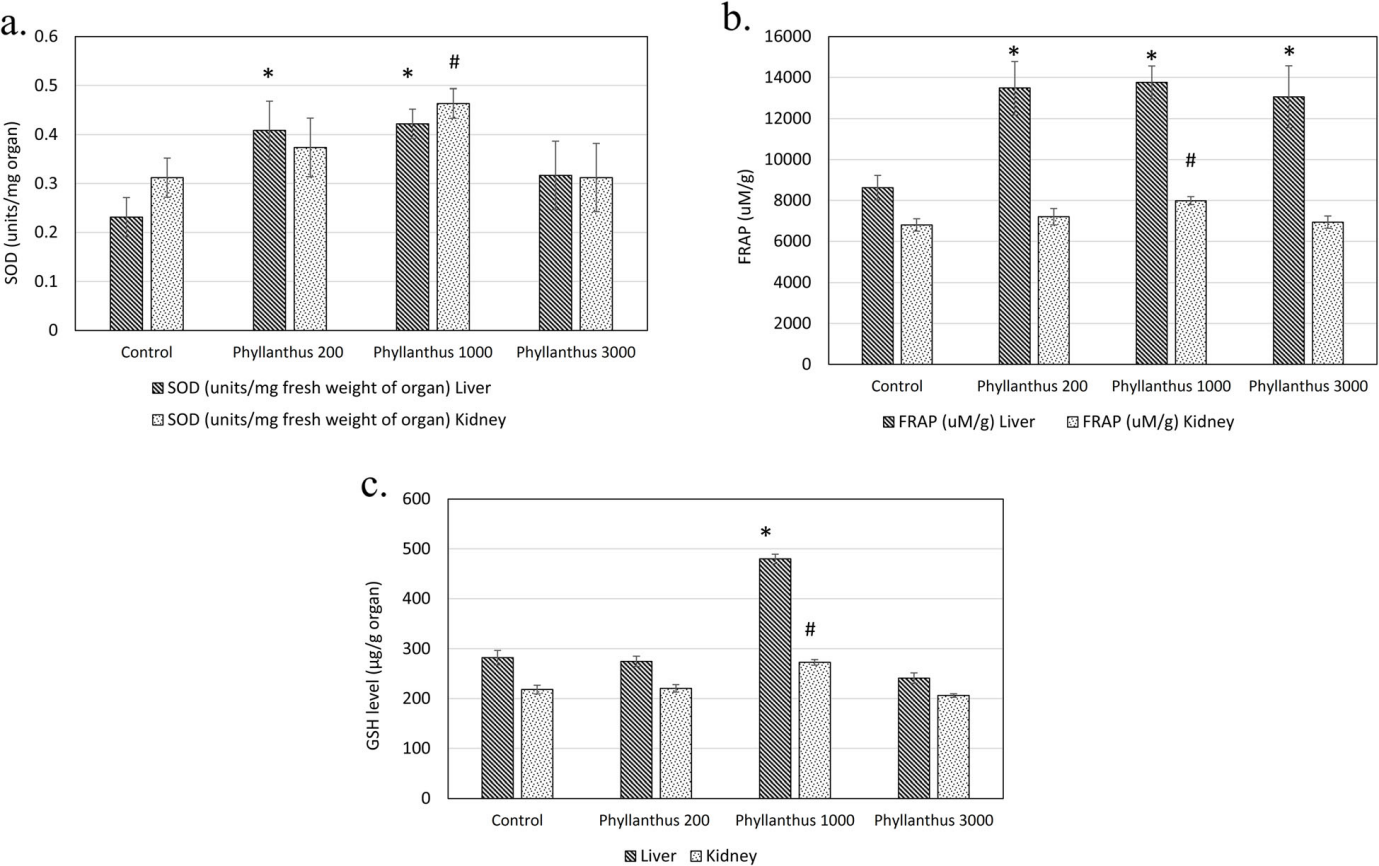
**a** SOD; **b** FRAP and **c** GSH level of liver and kidney of normal control and *Phyllanthus* (200, 1000 and 3000 mg/kg body weight) treated mice in sub-chronic toxicity study. *indicates a significant difference compared with the normal control group, *p* < 0.05

**Fig. 5:**
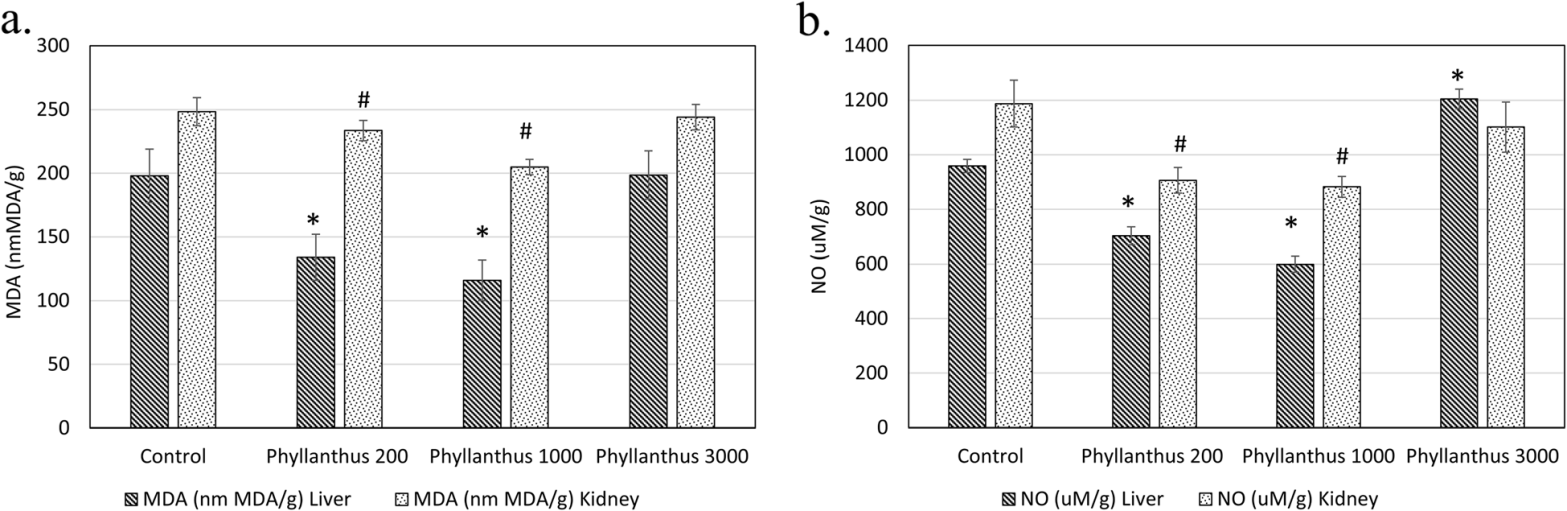
**a** MDA lipid peroxidation and **b** NO level of liver and kidney of normal control and *Phyllanthus* (200, 1000 and 3000 mg/kg body weight) treated mice in sub-chronic toxicity study. *indicates a significant difference compared with the normal control group, *p* < 0.05

SOD, GSH and FRAP antioxidant levels when compared to the normal control mice. Besides, this treatment also enhanced 1.48 folds, 1.17 folds and 1.24 folds of kidney SOD, GSH and FRAP antioxidant levels comparing to the normal control mice (Fig. 4a, b and c). In addition, MDA lipid peroxidation and NO level in the liver of mice treated with 1000 mg/kg body weight of *P. tenellus* extract were lower, ie 0.58 folds and 0.63 folds as compared to the normal control mice (Fig. 5a and b). Similar pattern was observed in the kidney of mice treated with 1000 mg/kg of extract, which recorded with 0.83 folds and 0.73 folds of MDA lipid peroxidation and NO level of the control mice (Fig. 5a and b). On the other hand, mice treated with 3000 mg/kg body weight of *P. tenellus* extract was reported with significantly 1.5 folds higher (*p* < 0.05) FRAP levels in the liver (Fig. 4b). In contrary with the lower dosage, highest concentration of *P. tenellus* extract was found with significantly 1.26 folds highest NO level in the liver (Fig. 5b).

## Discussion

*Phyllanthus* species have been widely used as a traditional medicine to treat urolithiasis, inflammation, diabetes and hepatitis. In comparison to other more popular *Phyllanthus* species, *P. tenellus* has not been exposed to elaborate scientific studies especially with respect to its bioactivity and toxicity studies [2]. In this previous study, a preliminary short-term acute toxicity study was performed and reported that up to 2500 mg/kg body weight of *P. tenellus* ethanol extract did not induce mortality in mice. However, slight agitation was observed in the mice treated with the highest dosage of the extract [2]. Similarly, in the present study, no cytotoxic effect was observed in NIH 3 T3 cells cultured with 200 μg/mL of the extract in vitro*.* In addition, no mortality was observed in this sub-chronic toxicity study of *P. tenellus* extract for all the tested concentrations. In addition, no significant changes of body weight and organ weight when comparing between normal control and *P. tenellus* extract treated mice were observed. These results indicate that *P. tenellus* extract did not induce sub-chronic toxicity up to 3000 mg/kg body weight. To further evaluate the effect of *P. tenellus* on liver and kidney, organ histological sections and serum biochemical markers were analyzed. At low dose of *P. tenellus* extract, no significant differences in the serum liver enzyme level compared to the normal control was observed. However, when the mice were treated with 1000 mg/kg body of *P. tenellus* extract, lower levels of serum liver enzyme and creatinine without any alterations of the liver and kidney histological sections were observed. This result reveal that a treatment of up to 1000 mg/kg body weight of extract on mice is considered safe as it did not cause any observable side effects to the liver and the kidney.

*P. tenellus* has been traditionally used for hepatitis [2]. Study by Srirama et al. [3] reported that methanolic extract of *P. tenellus* did not possess any in vitro hepatoprotective effect in contradiction to our results. In addition, the results of this study revealed that the hepatoprotective effect of *P. tenellus* could possibly be dose dependent. The highest concentration of *P. tenellus* extract (3000 mg/kg body weight) was observed with hepatocyte ballooning and increased serum liver enzyme markers. These results tend to support the finding of the acute toxicity study in the previous investigation [2]. With regard to this, consumption of high dosages of *P. tenellus* should be avoided regardless of it not causing mortality and morbidity of the treated mice.

Earlier phytochemical study on *P. tenellus* indicated that this plant contains high polyphenol and hydrosable tannin content [15], which are related to various bioactivities including antioxidant, antimicrobial [18] and immunomodulatory effects [19]. Similar effects are observed in this study where NIH 3 T3 cells treated with *P. tenellus* extract was observed with higher level of cellular GSH. Besides, mice treated with 1000 mg/kg body weight of *P. tenellus* extract exhibited higher levels of in-vivo antioxidant (FRAP, SOD and GSH) and were associated with lower levels of lipid peroxidation and nitric oxide accumulation both, in the liver and the kidney. The antioxidant activity is hypothesized to be attributed by the presence of hydrosable tannin particularly the catabolized ellagic acid (presumed to be Urolithin A) that has been detected in abundance and was the key compound in the extract [15]. Urolithin A is a bioactive secondary metabolite of complex ellagic acid [20]. Urolithin A has been reported to possess in vitro and in vivo antioxidant activity, thereby contributing to the protection of several reactive oxygen species (ROS) induced stress such as survival of neuronal cells under oxidative stress [21]. However, availability of Urolithin A is very much dependent on the presence of a specific intestinal microbe species from the *Eggerthellaceae* family which catabolizes ellagic acid to Urolithin A [20]. Pressurized hot water extraction was able to catabolize the ellagic acid to a metabolite believed to be Urolithin A in *P. tenellus* [15]. We believe that ‘Urolithin A’ may be one of the main contributors to the high in vivo antioxidant effect observed as this metabolite was detected in significantly high concentration compared to other metabolites [15].

In addition, intake of 200 and 1000 mg/kg body weight of the extract was also observed with lower levels of NO, which maybe attributed by the immunosuppression effect of Urolithin A [22]. On the other hand, a high dose of 3000 mg/kg of the extract was associated with significantly higher level of NO in the liver. Accumulation of NO may induce cellular stress to hepatocytes [23], and contribute to alteration of liver histological structure and serum liver enzyme levels. The mild hepatitis effect of high dosage of *P. tenellus* extract could be due to the high concentration of total tannin, similar to the in vivo adverse effects of high concentration of tannin containing *Mimosa pudica* root extract [24].

## Conclusions

The oral sub-chronic toxicity study of *P. tenellus* extract revealed that 1000 mg/kg body weight of the extract did not exhibit any toxic side effects. On the other hand, it enhanced the antioxidant levels in both the liver and kidney while suppressing the accumulation of NO. Although the 3000 mg/kg body weight of *P. tenellus* extract did not cause mortality or morbidity, mild toxicity was observed based on the preliminary findings of significant increase in serum liver enzymes associated with the accumulation of liver nitric oxide and hepatocytes ballooning degeneration. In short, pressurized hot water extraction of *P. tenellus* extract particularly at 1000 mg/kg has the potential to become an important therapeutic agent due to its high antioxidant capacity and NO- suppressing activity. However, more preclinical studies of the toxicity and bioactivities evaluation with the use of immunohistochemistry markers on the isolated hydrosable tannin particularly the Urolithin A are needed to validate the functions and the potential side effect of the hydrosable tannin in the *P. tenellus* extract.

## Data Availability

The datasets used and/or analysed during the current study are available from the corresponding author on reasonable request.

## Abbreviations

AEC: Animal Ethics Committee
ALP: Alkaline phosphatase
ALT: Alanine aminotransferase
AST: Aspartate aminotransferase
FRAP: Ferric-reducing ability plasma
GSH: Glutathione
LDH: Lactic acid dehydrogenase
MARDI: Malaysian Agriculture Research and Development Institute
MDA: Malonaldehyde
NO: Nitric oxide
ROS: Reactive oxygen species
SOD: Superoxide dismutase
